# Breastfeeding experiences during the COVID-19 pandemic in Spain:a qualitative study

**DOI:** 10.1186/s13006-022-00453-0

**Published:** 2022-02-22

**Authors:** Isabel Rodríguez-Gallego, Helen Strivens-Vilchez, Irene Agea-Cano, Carmen Marín-Sánchez, María Dolores Sevillano-Giraldo, Concepción Gamundi-Fernández, Concepción Berná-Guisado, Fatima Leon-Larios

**Affiliations:** 1 Maternal-fetal Clinical Management Unit, Genetics and Reproduction, Virgen del Rocío University Hospital, Red Cross Nursing University Centre, University of Seville, Seville, Spain; 2 Obstetrics and Gynecology, Virgen de las Nieves University Hospital, Granada, Spain; 3 Obstetrics and Gynecology, San Juan de la Cruz Hospital, Jaén, Spain; 4 Primary health care district, Huelva,, Spain; 5 Primary health care district, Osuna, Spain; 6 Obstetrics and Gynecology, Puerto Real University Hospital, Cádiz, Spain; 7 Primary health care district, Jaen, Spain; 8 Nursing Department, School of Nursing, Physiotherapy and Podiatry, University of Seville, Seville, Spain

**Keywords:** Breastfeeding, COVID-19, SARS-CoV-2, Breastfeeding support, Community health services, Public health, Primary health care, Lockdown

## Abstract

**Background:**

The pandemic caused by COVID-19 has affected reproductive and perinatal health both through the infection itself and, indirectly, as a consequence of changes in medical care, social policy or social and economic circumstances.

The objective of this study is to explore the impact of the pandemic and of the measures adopted on breastfeeding initiation and maintenance.

**Methods:**

A qualitative descriptive study was conducted by means in-depth semi-structured interviews, until reaching data saturation. The study was conducted between the months of January to May 2021. Participants were recruited by midwives from the Primary Care Centres of the Andalusian provinces provinces of Seville, Cádiz, Huelva, Granada, and Jaén. The interviews were conducted via phone call and were subsequently transcribed and analysed by means of reflexive inductive thematic analysis, using Braun and Clarke’s thematic analysis.

**Results:**

A total of 30 interviews were conducted. Five main themes and ten subthemes were developed, namely: Information received (access to the information, figure who provided the information), unequal support from the professionals during the pandemic (support to postpartum hospitalization, support received from Primary Health Care during the postpartum period), social and family support about breastfeeding (support groups, family support), impact of confinement and of social restriction measures (positive influence on breastfeeding, influence on bonding with the newborn), emotional effect of the pandemic (insecurity and fear related to contagion by coronavirus, feelings of loneliness).

**Conclusion:**

The use of online breastfeeding support groups through applications such as WhatsApp®, Facebook® or Instagram® has provided important breastfeeding information and support sources. The main figure identified that has provided formal breastfeeding support during this period was that of the midwife. In addition, the social restrictions inherent to the pandemic have exerted a positive effect for women in bonding and breastfeeding, as a consequence of the increase in the time spent at their homes and in the family nucleus co-living.

**Supplementary Information:**

The online version contains supplementary material available at 10.1186/s13006-022-00453-0.

## Background

On 11th March, 2020, the World Health Organization (WHO) declared a worldwide health emergency and pandemic due to exponential number of contagions caused by the new type of virus from the family Coronaviridae, COVID-19 [1, 2].

In Spain, the first recorded case dates back to 31st January, 2020, after conducting case-study epidemiological surveillance [3]. Due to the exponential increase in the number of infected people, hospitalizations and deaths, the Spanish government imposed social containment and isolation measures from 14th March, 2020 onwards, as a control and protection measure against the disease [4].

Most of the scientific research studies initially published were focused on assessing the effects of COVID-19 on the general population, reporting insufficient and unspecific data about the impact on specific populations, such as pregnant or lactating women [5–7]. At the beginning of the pandemic, information about this new coronavirus was limited: it was not known if it could be vertically transmitted from the mother to the baby in utero or after giving birth, through direct airways, inhalation, or breastfeeding (BF) [8]. This lack of information lead to the publication of misinformation and/or publication of contradictory information by institutions and caused the preventive interruption of the usual hospital practices, separating women from their newborns and partners during and after delivery, or even advising against breastfeeding [9]. Consequently, the pandemic caused by COVID-19 has affected reproductive and perinatal health both directly through the infection itself and, indirectly, as a consequence of changes in medical care, due to social policy or social and economic circumstances [10].

Currently, different international and national official entities state that breast milk is unlikely to be a source of infection and, therefore, it is not recommended to interrupt this practice, even in cases of confirmed maternal infection. Breastfeeding is thus advocated as the most adequate measure to protect the newborns’ health, encouraging that mothers continue breastfeeding as long as possible and in the exclusive modality, ideally during the first 6 months of life [11–13].

There is strong evidence that promotion of breastfeeding immediately after childbirth, skin-to-skin contact, mother-newborn rooming-in and support to initiate breastfeeding as soon as possible after childbirth, as well as community support, are favourable factors for breastfeeding success [14]. However, many of these aspects have been affected by the pandemic, both due to the adoption of new protocols in hospital centres and to the restrictive social measures imposed which have caused physical separation from family members, friends, and support networks [9].

Understanding how the pandemic caused by COVID-19 has affected the breastfeeding practice is crucial to obtain a global perspective of its impact on different aspects related to public health, but also to streamline the organization of perinatal health care in future waves or events. The objective of this study is to explore the impact of the pandemic on the measures adopted on breastfeeding initiation and maintenance.

## Methods

### Study design

This qualitative descriptive study was conducted following the Standards for Reporting Qualitative Research: A Synthesis of Recommendations (SRQR), a comprehensive checklist consisting of 21 items that covers the necessary components of study design which should be reported to improve the transparency in all aspects of qualitative research [15] (See additional file 1). The study was conducted between the months of January to May 2021.

### Participants, recruitment and study area

The eligible female participants were recruited in primary care health centres in Andalusia, Spain. Andalusia is an Autonomous Community divided into 8 provinces with a total population of 8,464,411 inhabitants (available data in 2020) [16], and a birth rate of 8.21 per 1000 population (2019) [17]. On July 1st 2020, the number of females of reproductive age in Andalusia was 1,966,790 [18].

Participants were recruited by midwives from the Primary Care Centres of the Andalusian provinces provinces of Seville, Cádiz, Huelva, Granada, and Jaén. “Snowball” intentional sampling was performed through these reference midwives, contacting the study profiles [19]. Women were mainly approached in the antenatal clinic by their midwife. Participants were identified via telephone after their midwives provided their telephone numbers. Then, through WhatsApp groups, women were contacted in order to check that they met the inclusion criteria. Finally, participants were recruited for the study and an appointment was scheduled to conduct the interview by phone.

The group of women participants was classified based on the criteria of homogeneity of newborn nutrition through breastfeeding and on a positive/negative diagnosis for the disease caused by COVID-19. The heterogeneity criteria were delivery before or after declaration of the SARS-CoV-2 pandemic and successful or interrupted breastfeeding.

### Inclusion criteria

Women over 18 years old and living in Andalusia (Spain) who had undergone delivery before or after pandemic declaration, with successful or interrupted breastfeeding during this period. Women with a positive COVID-19 diagnosis and delivery during the pandemic, with maternal or artificial lactation. Women able to communicate and understand the study requirements. Women who accept and sign the informed consent.

### Data collection

In-depth semi-structured interviews were carried out until reaching data saturation. For this purpose, an interview script was created (Table 1) to include the following items: sociodemographic profile, gynaecological-obstetric aspects, perinatal data, breastfeeding, information on breastfeeding and COVID-19, impact of the COVID-19 pandemic on breastfeeding, breastfeeding support and experience with breastfeeding.

**Table 1:** Interview

Interview script	
Themes	Sample questions
**Sociodemographic data**	What’s your name? How old are you? What’s your nationality? In which province do you live? What’s your marital status? Do you have a partner? What’s your schooling level? What’s your profession? Do you work? Are you a freelancer or an employee?
**Gynaecological-obstetric data**	How many pregnancies have you had? Have they been normal o caesarean deliveries? How many children do you have? Do you have any previous breastfeeding experience? How long have you been breastfeeding? What was the reason for abandonment? When was your last delivery? What type of delivery was it? How do you remember your experience? Did you, your companion or the NB undergo any diagnostic test for COVID-19 when entering or staying in the hospital? Was there mother-NB separation during hospitalization due to the protocols implemented as a consequence of COVID-19? How would you describe this situation?
**Perinatal data**	What’s the gender of your newborn? Please indicate his/her weight and height. Was admittance to Neonatology necessary?
**Breastfeeding**	How did you decide to feed your son/daughter? What type of breastfeeding were you offering at hospital discharge? Did you have any difficulty during this period? What type of breastfeeding are you offering now? In case of breastfeeding abandonment, what was the reason?
**COVID-19 and Breastfeeding**	What information did you have about this topic? What did they tell you? Where did you get the information? Did the information condition your intention to breastfeed? What different measures did you adopt for breastfeeding and to avoid contagion in your baby? Did you feel insecurity or fear infecting your baby?
**Impact of the pandemic**	Who did you live with during the confinement period? Do you consider that the pandemic or confinement situation affected your breastfeeding plans? How? What was your initial idea? What happened? Did you find difficulties having a successful breastfeeding during the pandemic/confinement? Mention them. How have you felt during this period?
**Breastfeeding support**	Which do you consider were your support options during this period to be successful with your breastfeeding? In case of previous attendance to breastfeeding support groups, how did they organize during this period? How did they contact them? Did you find practicalities from the health services?
**Breastfeeding experience**	How would you describe your experience with breastfeeding?

Source: Elaborated by the authors

### Data collection methods

Due to the current public health situation caused by COVID-19 and lockdown restrictions, interviews were conducted online via phone calls. Thus, calls were scheduled and arranged with prior informed consent. The main researcher tried to maintain a comfortable environment for all participants to favour the interviewee’s privacy. The research team used Lincoln and Guba’s criteria to establish the trustworthiness of the study. These authors describe that trustworthiness in a study is determined by credibility, dependability, reflexivity, transferability, and confirmability [20]. In this way, interviews were conducted by the main researcher of the study (IRG). They were recorded and transcribed in the same language by the first author, a PhD candidate not involved in women’s care (IRG) (reflexivity). Subsequently, the transcribed interviews were sent to some participants at random (credibility and dependability) for data feedback and to ensure that the meaning expressed during the interviews was maintained. Finally, during the process of data analysis all the information was triangulated by the study researchers to enhance validity and confirmability.

### Data analysis

All the data were analysed by two different researchers (FLL-IRG). IRG is a PhD candidate not involved in women’s care, as well as FLL, who is a PhD researcher with previous experience in qualitative research. The interviews were analysed by means of reflexive inductive thematic analysis. The transcribed interviews were examined using this approach to identify their meanings, and the information was extracted and categorized into themes and subthemes.

Firstly, the research team became familiar with the data and performed the coding. The interviews were independently analysed by the two researchers (FLL-IRG), and the subsequent interpretations were compared by the research team. Additionally, regular analytical sessions were held with the research team to generate, review and name of themes. Subsequently, the two researchers held regular meetings during the rest of the analysis process.

For this purpose, a reactive, iterative systematic process was accomplished, attending to the phases proposed by Braun and Clarke [21, 22]. The structural analysis of the different parts, reflexivity, and the semantic and pragmatic triangulation by the researchers allowed us to confer quality and rigor to this study [20]. The quotes given in the results section were included for their representativeness and selected after verifying their accuracy. The analysis was carried out in Spanish and the quotes were later translated into English and reviewed by a professional native translator with credentials and experience in the field, not directly involved in the data collection process.

### Ethical consideration

This study has been conducted in strict compliance with the ethical principles of the Declaration of Helsinki (1964), including the informed consent request for the participants. Participation in the project was voluntary, as well as the signing of participation request. A verbal informed consent was provided to every participant in this study.

The study has been laid out according to Spanish regulation act No. 14/2007 of July 3rd regarding biomedical research, complying with the study suitability requirements and with the procedure regarding the study objectives. All patient-related data collected for this study will be treated according to the Spanish Organic Law on Protection of Personal Data and Guarantee of Digital Rights (Spanish Organic Law 3/2018).

## Results

A total of 42 women who met the inclusion criteria were recruited. A total of 30 interviews were finally conducted until reaching theoretical data saturation. The first step involved making the phone calls in order to arrange the interviews by recruitment order. Some women were unable to be contacted or were unavailable and therefore they could not be interviewed. The interviews approximately lasted between 35 and 45 min. The participants’ age group corresponds to that from 19 to 43 years old. All participants had Spanish nationality, they were married or had a steady partner, high school/university studies and employed, mostly. The sociodemographic.

profile and data related to breastfeeding in the women interviewed can be seen in detail in Table 2.

**Table 2:** Participant’s characteristics

Interviewee	Age, years	Nationality	Marital status	Maternal Educational level	Occupation	Parity	Previous breastfeeding experience	Infant feeding (first six months)	Age of the infants
E1	35	Spanish	Married	College degree	Unemployment	Multiparous	Yes	Exclusively breastfeeding	Three months
E2	25	Spanish	Married	High School	Employed	Primiparous	–	Nonexclusively breastfeeding	Two months
E3	31	Spanish	Married	College degree	Employed	Primiparous	–	Exclusively breastfeeding	Four months
E4	38	Spanish	Married	College degree	Unemployment	Multiparous	Yes	Exclusively breastfeeding	Eight months
E5	35	Spanish	Married	High School	Self-employed	Multiparous	Yes	Exclusively breastfeeding	Three months
E6	33	Spanish	In a relationship	High School	Employed	Primiparous	–	Exclusively breastfeeding	Ten months
E7	38	Spanish	Married	College degree	Employed	Primiparous	–	Nonexclusively breastfeeding	Four months
E8	34	Spanish	Married	High School	Employed	Multíparous	Yes	Exclusively breastfeeding	Eleven months
E9	39	Spanish	Married	High School	Employed	Multíparous	Yes	Exclusively breastfeeding	Ten months
E10	33	Spanish	Married	High School	Unemployment	Multiparous	No	Formula feeding	Two months
E11	30	Spanish	In a relationship	High School	Self-employed	Primiparous	–	Exclusively breastfeeding	Six months
E12	29	Spanish	Married	High School	Self-employed	Multiparous	Yes	Exclusively breastfeeding	Nine months
E13	33	Spanish	Married	College degree	Employed	Primiparous	–	Exclusively breastfeeding	Nine months
E14	42	Spanish	In a relationship	High School	Employed	Primiparous	–	Exclusively breastfeeding	Ten months
E15	19	Spanish	In a relationship	High School	Employed	Primiparous	–	Exclusively breastfeeding	Ten months
E16	33	Spanish	Married	College degree	Employed	Primiparous	–	Exclusively breastfeeding	Eleven months
E17	20	Spanish	In a relationship	High School	Unemployment	Multiparous	Yes	Nonexclusively breastfeeding	Seven months
E18	38	Spanish	In a relationship	High School	Employed	Primiparous	–	Nonexclusively breastfeeding	Five months
E19	32	Spanish	In a relationship	High School	Employed	Primiparous	–	Exclusively breastfeeding	Four months
E20	27	Spanish	In a relationship	High School	Employed	Primiparous	–	Nonexclusively breastfeeding	Eight months
E21	21	Spanish	In a relationship	High School	Unemployment	Primiparous	–	Nonexclusively breastfeeding	Ten months
E22	27	Spanish	Married	High School	Employed	Multiparous	Yes	Exclusively breastfeeding	Ten months
E23	35	Spanish	In a relationship	College degree	Employed	Primiparous	–	Nonexclusively breastfeeding	Eleven months
E24	38	Spanish	In a relationship	High School	Unemployment	Primiparous	–	Formula feeding	Five months
E25	38	Spanish	In a relationship	High School	Employed	Primiparous	–	Nonexclusively breastfeeding	Six months
E26	38	Spanish	Married	High School	Self-employed	Primiparous	–	Exclusively breastfeeding	Eleven months
E27	43	Spanish	Divorced	College degree	Employed	Primiparous	–	Exclusively breastfeeding	Nine months
E28	26	Spanish	In a relationship	High School	Employed	Primiparous	–	Exclusively breastfeeding	Six months
E29	35	Spanish	In a relationship	Less than a high school degree	Employed	Multiparous	No	Exclusively breastfeeding	Ten months
E30	33	Spanish	In a relationship	High School	Unemployment	Multiparous	Sí	Exclusively breastfeeding	Six months

Source: Elaborated by the authors

During the data analysis process, five main interrelated themes and ten subthemes were developed and interpreted by the researchers (Table 3).

**Table 3:** Themes and subthemes

Themes	Subthemes
Information received	Access to the information
	Figure who provided the information
Unequal support from the professionals during the pandemic	Support during postpartum hospitalization
	Support received from Primary health Care during postpartum
Social and family support on breastfeeding	Breastfeeding support groups
	Family support for breastfeeding
Impact of confinement and of the social restriction measures	Positive influence on breastfeeding
	Influence on bonding with the newborn
Emotional effects of the pandemic	Insecurity and fear related to contagion by coronavirus
	Feelings of loneliness

Source: Elaborated by the authors

### Information received

#### Access to the information

The interviewees expressed lack of unified information from the health services in relation to the effect of coronavirus on pregnancy, delivery and breastfeeding. The main information sources used to access this knowledge were Internet searches and WhatsApp® groups headed by health professionals, relying on peer-support and based on other women’s experiences.

*“I’ve been reading and looking at things on the Internet, everything I saw was that it was better to breastfeed than not to breastfeed. She’s going to be more protected with breast milk than with the formula” (E4).*

*“The midwife sent us some documents through the WhatsApp breastfeeding support group” (E6).*

*“I didn’t have much information about breastfeeding and COVID-19, what I’d read on the Internet and what my midwife sent us” (E10).*

*“There was a lot of disinformation, I searched the Internet...but as everything was so recent, there was not much” (E12).*

#### Figure who provided the information

The reference midwife working in the health centre was the main figure used as information source during the pandemic, making use of alternative resources to the usual appointments, such as WhatsApp® groups with pregnant and puerperal women:

*“Our midwife always kept us informed, she created a WhatsApp group with those who were going to have a baby and those who have had one during the pandemic months and so we contacted her” (E9).*

*“The midwife sent us some documents through the breastfeeding support group” (E6).*

*“The information our midwife gave us was that it was important to breastfeed for the antibodies and that it helped for the baby not to get infected by COVID” (E11).*

### Unequal support from the professionals during the pandemic

#### Support received during postpartum hospitalization

The women identified lack of information and support by the professionals in the first postpartum days, during the hospitalization period, expressed as follows:

*“At no time did they tell us anything, we breastfed the child and that’s it” (E1).*

*“There was a little bit of everything among the health professionals, there was the one who allowed “the help” and the one that said that everything was going to be alright if you breastfed” (E6).*

*“There’s really very little information on breastfeeding in the hospital, nobody who comes and explains anything, or even attentive of what happens to the mother and the baby” (E11).*

*“In the hospitals, there are many nurses who are not linked to maternal breastfeeding and they’re already asking you if you want a little help” (E4).*

*“I didn’t find support in the hospital, they came and put it to breastfeed, but it was them that made it latch and I didn’t learn” (E12).*

#### Support received from primary health care during the postpartum period

Most of the interviewees emphasized that the support they received from the reference midwife working in Primary Care during the postpartum period was of good quality, although they also expressed some difficulties related to the telematic consultations that did not help solve problems associated with breastfeeding, which would have needed an assessment of the difficulty.

*“My major supports were the midwife and another mother from the breastfeeding support group. It is thanks to my midwife that I go on with breastfeeding, I would’ve abandoned it if I wasn’t for her. Every mother deserves a midwife like her and a breast feeding support group.” (E6).*

*“I wasn’t able to make a normal appointment with the midwife, I’ve had mastitis for a long time … As they don’t see you, you’re alone” (E7).*

*“Everything has been over the phone, and there are things that you need to see in person” (E19).*

Likewise, some women expressed lack of support by other reference professional figures:

*“I didn’t find support, certainly not in the health centre” (E1).*

“*There’s very little updating on the part of the professionals, if it was for the paediatrician, I would’ve abandoned breastfeeding a long time ago, thank God that my midwife advises me” (E19).*

### Social and family support on breastfeeding

#### Breastfeeding *support groups*

The women identified the breastfeeding support groups as one of the main support options during this experience. The organization of these groups has undergone a change in their format with the pandemic: from the usual in-person meetings, most are now held virtually through WhatsApp®, or social networks such as Facebook® and Instagram®. Women also identified it as a useful resource for their experience:

*“I’ve stayed in touch with the same breastfeeding support group as with my first baby, they’ve helped me a lot. When I’ve had doubts, they solved everything via WhatsApp or they called me, they’ve been my main support” (E30).*

*“I got the information from the internet mainly, because the major problem pregnant women faced then was the suspension of birth preparation workshops and face-to-face support groups. It was via Facebook that I joined a breastfeeding support group” (E27).*

*“Instagram really saved me, because I gave birth and once, I was discharged, we were already under lockdown. Instagram was quite a lifesaver, the people’s comments, the instructions from the midwives and the breastfeeding counsellors” (E16).*

*“I have a WhatsApp breastfeeding support group from the town, my midwife contacted me with them. It’s a group where they solve you doubts anytime … In addition to support, it’s peace of mind. If you have any doubt, you have someone to advise you and explain anytime” (E11).*

#### *Family support for* breastfeeding

The women identified their family members as important figures in the support received during breastfeeding; they especially highlight their partners as main providers of this support. The support that these women acknowledge is of the informative, emotional and practical help type, by completing the other house chores:

*“My partner has been the main support, I breastfed, but he cheered me” (E15).*

*“My partner has been of great help; he’s always taken care of everything that was not breastfeeding” (E27).*

*“My partner has been my main support, helping in the house and also looking for information if I was exhausted” (E19).*

Likewise, the women recognized other people near them as support figures, or even expressed their losing this support due to the pandemic situation experienced. This support was mainly provided by other women near them with previous experience regarding motherhood:

*“My cousin has been giving me tips, she’s had two girls and has breastfed for a long time” (E29).*

*“It was thanks to my mother, who told me not to get tired and to breastfeed a lot, that the girl latched” (E28).*

*“The female support network we normally have are mother, sisters, aunts … It has also been lost nowadays” (E27).*

*“My sister has been my main support, she’s also a mother and has previous experience” (E6).*

Likewise, the women acknowledge that the influence exerted by the figures near them may not always favour breastfeeding and that, in view of some difficulties encountered, the opinions and lack of support received can be an obstacle and a reason to abandon breastfeeding:

*“In relation to my parents, perhaps the way to tell me how it was hasn’t been the best, they told me things that the baby was not satisfied and that I had to give him the bottle. You feel pretty bad, as if you left your child hungry … You even doubt if he really needs the bottle or not, I didn’t feel understood” (E2).*

*“People’s comments are pretty influential but, in the end, your maternal instinct wins over and you see your child happy breastfeeding” (E6).*

*“The family is 100% influential because, for example in my case, it didn’t work the first time and they saw me exhausted, they told me things … and of course I’ve already left it. It’s very important that they support you” (E8).*

*“They had given the bottle and expected me to do so as well, I’ve fallen short of their expectation not doing it” (E12).*

### Impact of confinement and of the social restriction measures

#### Positive influence on breastfeeding

The women identified the experience of confinement and social restriction measures as a positive impact on their breastfeeding experience. They stated that staying more time in their homes, caring for the newborn and not having any visits represented an element that favoured breastfeeding initiation and maintenance.

*“I believe that social isolation has favoured breastfeeding, in the first week, it gained 500 g instead of losing weight” (E4).*

*“If the situation had been normal as before the virus, that you could go out and come in, I believe that I would’ve abandoned it much earlier. ( …*) *I’m a slave to the two children and I couldn’t go out anywhere” (E7).*

*“This breastfeeding has been a calmer experience, as nobody came here the girl has latched more calmly, all the time she wanted, with no external noises and nobody bothering her” (E9).*

*“For my girl, confinement has been the best thing ever, because she was totally demanding, no one has interrupted us because they knew they couldn’t come” (E14).*

#### Influence on bonding with the newborn

On the other hand, the women also stated that this time alone with the family nucleus, without external interference or interruptions, was also identified as positive in the experience of creating the new family and in the bond that is created with the newborn:

*“I’ve felt a stronger bond among the four of us for being all the time together, it’s been gratifying” (E4).*

*“I was focused on my child, I had nothing else to think about or do, I believe that it had a positive effect” (E6).*

*“Even if it sounds bad, confinement has been a benefit, actually, because we haven’t had any visits, it’s been only us the family nucleus and we’ve all benefited in that sense” (E8).*

“*Being just the two of us, with no visitors, I was fully engaged in childcare and my daughters, resting and recovering myself. I was only focused on what was important, on that connection and on being calm” (E12).*

*“I believe that, at the level of family adaptation and with breastfeeding, confinement has done us pretty good because nobody bothered us. We assembled as a family naturally, without interruptions” (E16).*

### Emotional effects of the pandemic

#### Insecurity and fear related to contagion by coronavirus

The interviewees expressed feelings related to fear and insecurity towards contagion by this new coronavirus, as well as for the possibility of infecting their children, reason why they maximized the hygiene measures to avoid virus transmission:

*“It is frightening that you get infected and can infect others” (E13).*

*“My main fear is for me to be asymptomatic and pass it on to him” (E6).*

*“Always with fear, without taking our masks off, washing our hands a lot” (E11).*

*“Mood has been very different than before, the truth is that very bad, a lot of time alone … I didn’t want anybody to come for fear of contagion” (E10).*

*“It’s affected be me a lot, wherever I go or whoever passes by me … everything scares me” (E2).*

#### Feelings of loneliness

The women expressed feelings of loneliness and sorrow related to confinement and to isolation from important people near them:

*“In my case, because it’s been the third, if it had been the first and confined it would’ve been three times worse, because being alone, without experience …*” *(E8).*

*“We were fine, on the one hand, easy at home … But also a little sad at the family level, because the grandparents haven’t met her yet” (E9).*

*“The greatest influence of the pandemic is not being able to have my family here, in my house” (E10).*

*“Not being able to share those moments has been tough, not being able to get a hug from your mother or your sisters” (E26).*

*“Lockdown has marked me for real. I’m sure I had postnatal depression. It was not just that I could not go out freely, but also the fear, and being unable to see my parents … It’s been horrible from the mood perspective, the sensation of isolation and loneliness” (E27).*

*“It’s been tough, I now think about it and I even get emotional … I’ve had a really bad time. There were days that I was fed up crying and didn’t want to talk to anybody. I also felt sad that my child was always inside the house without seeing anybody, not getting a ray of light, anything” (E6).*

## Discussion

This study is pioneering in Spain and Andalusia, in relation to knowing the experiences related to breastfeeding during the exceptional pandemic situation, from a qualitative perspective. This study contributes valuable results about the experiences and resources women had regarding breastfeeding during the pandemic and lockdown. These findings can help to improve the experiences of other mothers, babies, and families in similar situations in the future. Even during emergency situations such as a pandemic and lockdown, women’s rights must be respected during pregnancy, delivery, and puerperium care.

Access to the information related to breastfeeding and COVID-19 has been extensively produced through the Internet and the social networks, recognizing these resources as sources of health-related information [23, 24]. Likewise, the women have considered midwives as safe sources of information based on scientific evidence, valuing their informative, practical and emotional support, acknowledging them as invaluable support figures during breastfeeding, and who have enabled their experience to be longer and pleasant, according to what is described in the literature [25].

The experiences in relation to breastfeeding during the pandemic have been diverse. On the one hand, some women reported that the confinement measures exerted a positive effect on their experience in relation to breastfeeding, as they allowed them more time with their children; however, another important number of women state that lack of support, limited information, and reduction in the number of appointments with the professionals exerted a negative influence on their practice, in line with other international studies recently published [26–29].

Among the main social support strategies, participants mentioned breastfeeding support groups as a useful resource, according to some research studies [30–32]. During this period, most of them have been organized online via WhatsApp® or Facebook®, being equally effective when compared to the classic in-person format, as also endorsed by some authors [33, 34].

As mentioned by the participants, the support provided by the health professionals was controversial: an important number of participants stated that it was of good quality and satisfactory to solve their breastfeeding problems, especially the support provided by the Primary Care midwife. In relation to what is described in the literature, access to support and the positive perception thereof in the first postpartum months were related to better exclusive breastfeeding rates at 6 months of life [35, 36]. On the other hand, another important group of women identified the support received during the first postpartum days as deficient, especially during hospitalization, which was described as a factor related to early breastfeeding abandonment [37, 38].

In relation to the family support provided, the special situation with mobility restriction and social isolation measures favoured that most of the women developed their breastfeeding experience separated from this support network, thus expressing its absence. This informal support network has been previously described by various authors as a complex network but, at the same time, with a potential impact on breastfeeding and with significant influence on breastfeeding initiation and maintenance in the short- and long-term, even favouring its extension beyond 6 months of life, after the mother is reinstated to her work environment [39–41]. Within the family environment, other female family members or the woman’s partner are usually the figures that provide most of this support, with the findings of this research being similar [42–44].

Likewise, the pandemic has exerted an important psychological effect, with the possibility of not only being related to negative mental health outcomes but also to breastfeeding success or failure [45]. Many women expressed concerns related to the impact of COVID-19 on their children and on themselves, as well as they also experienced feelings of loneliness and sorrow related to social isolation, in line with recent publications [46, 47], where the expression of depressive symptoms has also been related to breastfeeding.

### Limitations

This study presents two limitations: on the one hand, the impossibility of conducting in-person interviews, so as to ensure a better atmosphere of trust and warmth. For the same reason, control measures and coronavirus transmission, focus groups could not be conducted to complete the study and obtain in-depth information. However, the telematic data collection approach has become increasingly popular and necessary during the pandemic.

Another important limitation has been the likely participation of those women who have had very positive or very negative experiences during the pandemic, as well as of older women and with higher schooling levels, as in other similar studies, leaving aside minority groups that are also part of society.

## Conclusions

The pandemic has supposed important implications and challenges associated with adaptation to motherhood and the support provided to breastfeeding. In the Primary Health Care scope, new resources have been used to favour communication with women, such as telematic appointments or WhatsApp® groups, especially those headed by midwives. Together with the breastfeeding support groups organized via this modality or through social networks such as Facebook or Instagram, they have exerted a positive influence on the women’s breastfeeding experience during this period.

The social restrictions inherent to the pandemic have exerted a positive effect for the women in bonding and breastfeeding, as a consequence of the increase in the time spent at their homes and in the family nucleus co-living. However, there is also a negative perception associated with these restrictions, especially regarding family support.

### Implications and recommendations

The resources implemented during the pandemic might suppose a valid alternative in those situations in which in-person support cannot be provided, with a view to promoting and maintaining breastfeeding.

We recommend the use of the new technologies as a means for scientific dissemination and as a complementary support method during breastfeeding initiation and maintenance, mainly the online groups headed by midwives, where peer support can also be offered.

## Supplementary Information

**Additional file 1.** Standards for Reporting Qualitative Research.

## Data Availability

The datasets used and/or analysed during the current study are available from the corresponding author on reasonable request.

## Abbreviations

BF: Breastfeeding
SRQR: Standards for Reporting Qualitative Research: A Synthesis of Recommendations
WHO: World Health Organization

## References

[1] de Sanidad M. Centro de Coordinación de Alertas y Emergencias Sanitarias: Enfermedad por nuevo Coronavirus, COVID-19. https://www.mscbs.gob.es/profesionales/saludPublica/ccayes/alertasActual/nCov/documentos/Informacion_inicial_alerta.pdf. Accessed 15 May 2020

[2] WHO. WHO Director-General’s Opening Remarks at the Media Briefing on COVID-19 2020. https://www.who.int/director-general/speeches/detail/who-director-general-s-opening-remarks-at-the-media-briefing-on-covid-19%2D%2D-11-march-2020 .Accessed 15 May 2020.

[3] Red Nacional de Vigilancia Epidemiológica. Primeros casos investigados en España por COVID-2019. Informe COVID-2019 n° 1. 2020. Instituto de Salud Carlos III. https://www.isciii.es/QueHacemos/Servicios/VigilanciaSaludPublicaRENAVE/EnfermedadesTransmisibles/Documents/INFORMES/Informes%20COVID-19/Informe%20COVID-19.%20N%C2%BA%201_11febrero2020_ISCIII.pdf#search=primer%20caso%20covid%2D19. Accessed 15 May 2020.

[4] Agencia Estatal. Boletín Oficial del Estado. Real Decreto 463/2020, de 14 de marzo, por el que se declara el estado de alarma para la gestión de la situación de crisis sanitaria ocasionada por el COVID-19. https://www.boe.es/buscar/doc.php?id=BOE-A-2020-3692. Accessed 15 may 2020.

[5] Li Q, Guan X, Wu P, Wang X, Zhou L, Tong Y (2020). Early transmission dynamics in Wuhan, China, of novel coronavirus-infected pneumonia. N Engl J Med.

[6] Huang C, Wang Y, Li X, Ren L, Zhao J, Hu Y (2020). Clinical features of patients infected with 2019 novel coronavirus in Wuhan, China. Lancet.

[7] Wang D, Hu B, Hu C, Zhu F, Liu X, Zhang J (2020). Clinical characteristics of 138 hospitalized patients with 2019 novel coronavirus-infected pneumonia in Wuhan. China JAMA.

[8] Juan J, Gil MM, Rong Z, Zhang Y, Yang H, Poon LC (2020). Effect of coronavirus disease 2019 (COVID-19) on maternal, perinatal and neonatal outcome: systematic review. Ultrasound Obstet Gynecol.

[9] Renfrew MJ, Cheyne H, Dykes F, Entwistle F, McGuire W, Shenker N, et al. Optimising mother-baby contact and infant feeding in a pandemic. Rapid review. https://www.rcpch.ac.uk/sites/default/files/2020-06/optimising_mother_baby_contact_and_infant_feeding_in_a_pandemic_version_2_final_24th_june_2020.pdf. Accessed 15 may 2020.

[10] Kotlar B, Gerson E, Petrillo S, Langer A, Tiemeier H. The impact of the COVID-19 pandemic on maternal and perinatal health: a scoping review. Reprod Health. 2021;18(10). 10.1186/s12978-021-01070-6. 10.1186/s12978-021-01070-6 PMC7812564 33461593

[11] CDC. Coronavirus disease and breastfeeding. https://www.cdc.gov/breastfeeding/breastfeeding-special-circumstances/maternal-or-infant-illnesses/covid-19-and-breastfeeding.html Accessed 20 may 2020.

[12] Organización Mundial de la Salud. Preguntas frecuentes: Lactancia materna y COVID-19 Para trabajadores de la salud. https://www.who.int/docs/default-source/coronaviruse/breastfeeding-covid-who-faqs-es-12may2020.pdf?sfvrsn=f1fdf92c_. Accessed 15 may 2020.

[13] Iniciativa para la Humanización de la asistencia al nacimiento y lactancia. Lactancia materna ante la pandemia de Coronavirus COVID-19. Información para los profesionales que atienden familias con niños y niñas pequeños. https://www.ihan.es/wp-content/uploads/CORONAVIRUS-POSICION-IHAN-v6.pdf. Accessed 15 may 2020.

[14] Renfrew MJ, McCormick FM, Wade A, Quinn B, Dowswell T (2012). Support for healthy breastfeeding mothers with healthy term babies. Cochrane Database Syst Rev.

[15] Tong A, Sainsbury P, Craig J (2007). Consolidated criteria for reporting qualitative research (COREQ): A 32- item checklist for interviews and focus groups. International J Qual Health Care.

[16] Instituto Nacional de Estadística. Oficina Estadística Española. Registro INE; 2020. https://www.ine.es/jaxiT3/Datos.htm?t=2915#!tabs-tabla Acessed 20 May 2021

[17] Instituto Nacional de Estadística. Oficina Estadística Española. Registro INE; 2019. https://www.ine.es/jaxiT3/Datos.htm?t=1433#!tabs-tabla Acessed 20 May 2020

[18] Instituto Nacional de Estadística (INE). Series detalladas desde 2002. Población residente por fecha, sexo, grupo de edad y nacionalidad (agrupación de países). Oficina Estadística Española. Registro INE; 2020. https://www.ine.es/jaxiT3/Tabla.htm?t=9689 Accesed 20 May 2020

[19] Babbie E. The logic of sampling. In The Practice of Social Research. Howard Higher Education editor. Belmont. 2004:184–5.

[20] Korstjens I,Albine Moser A.Series: Practical guidance to qualitative research. Part 4: trustworthiness and publishing, Eu J Gen Pract 2018; 24(1):120–124. 10.1080/13814788.2017.1375092. 10.1080/13814788.2017.1375092 PMC8816392 29202616

[21] Braun V, Clarke V (2006). Using thematic analysis in psychology. Qual Res Psychol.

[22] Souza LK (2019). Pesquisa com análise qualitativa de dados: conhecendo a Análise Temática. Arq.bras.psicol..

[23] Zhao Y, Zhang J (2017). Consumer health information seeking in social media: a literature review. Health Info Libr J.

[24] Venegas-Vera AV, Colbert GB, Lerma EV (2020). Positive and negative impact of social media in the COVID-19 era. Rev Cardiovasc Med.

[25] Dagla M, Mrvoljak-Theodoropoulou I, Vogiatzoglou M, Giamalidou A, Tsolaridou E, Mavrou M (2021). Association between breastfeeding duration and long-term midwifery-led support and psychosocial support: outcomes from a Greek non-randomized controlled perinatal health intervention. Int J Environ Res Public Health.

[26] Ceulemans M, Verbakel JY, Van Calsteren K, Eerdekens A, Allegaert K, Foulon V (2020). SARS-CoV-2 infections and impact of the COVID-19 pandemic in pregnancy and breastfeeding: results from an observational study in primary care in Belgium. Int J Environ Res Public Health.

[27] Brown A, Shenker N (2021). Experiences of breastfeeding during COVID-19: lessons for future practical and emotional support. Matern Child Nutr.

[28] Vazquez-Vazquez A, Dib S, Rougeaux E, Wells JC, Fewtrell M.S. The impact of the Covid-19 lockdown on the experiences and feeding practices of new mothers in the UK: preliminary data from the COVID-19 new mum study. Appetite. 2021;156:104985. 10.1016/j.appet.2020.104985. 10.1016/j.appet.2020.104985 PMC7538871 33038477

[29] Latorre G, Martinelli D, Guida P, Masi E, De Benedictis R, Maggio L (2021). Impact of COVID-19 pandemic lockdown on exclusive breastfeeding in non-infected mothers. Int Breastfeed J.

[30] Rodríguez-Gallego I, Leon-Larios F, Corrales-Gutierrez I, González-Sanz JD (2021). Impact and effectiveness of group strategies for supporting breastfeeding after birth: a systematic review. Int J Environ Res Public Health.

[31] van Dellen SA, Wisse B, Mobach MP, Dijkstra A (2019). The effect of a breastfeeding support programme on breastfeeding duration and exclusivity: a quasi-experiment. BMC Public Health.

[32] Quinn EM, Gallagher L, de Vries J (2019). A qualitative exploration of breastfeeding support groups in Ireland from the women's perspectives. Midwifery..

[33] Niela-Vilén H, Axelin A, Melender HL, Löyttyniemi E, Salanterä S (2016). Breastfeeding preterm infants—a randomized controlled trial of the effectiveness of an internet-based peer-support group. J Adv Nurs.

[34] Robinson A, Lauckner C, Davis M, Hall J, Anderson AK. Facebook support for breastfeeding mothers: a comparison to offline support and associations with breastfeeding outcomes. Digit. Health. 2019;5. 10.1177/2055207619853397. 10.1177/2055207619853397 PMC6560800 31218076

[35] Gianni ML, Bettinelli ME, Manfra P, Sorrentino G, Bezze E, Plevani L (2019). Breastfeeding difficulties and risk for early Bbeastfeeding cessation. Nutrients..

[36] Kim SK, Park S, Oh J, Kim J, Ahn S (2018). Interventions promoting exclusive breastfeeding up to six months after birth: A systematic review and meta-analysis of randomized controlled trials. Int J Nurs Stud.

[37] Feenstra MM, Jørgine Kirkeby M, Thygesen M, Danbjørg DB, Kronborg H. Early breastfeeding problems: a mixed method study of mothers' experiences. Sex Reprod Healthc. 2018;(16):167–74. 10.1016/j.srhc.2018.04.003. 10.1016/j.srhc.2018.04.003 29804762

[38] Ceulemans M, Hompes T, Foulon V (2020). Mental health status of pregnant and breastfeeding women during the COVID-19 pandemic: a call for action. Int J Gynecol Obstet.

[39] Emmott EH, Page AE, Myers S (2020). Typologies of postnatal support and breastfeeding at two months in the UK. Soc Sci Med.

[40] Tchaconas A, Keim SA, Heffern D, Adesman A (2018). Pediatric care providers, family, and friends as sources of breastfeeding support beyond infancy. Breastfeed Med.

[41] Ratnasari D, Paramashanti BA, Hadi H, Yugistyowati A, Astiti D, Nurhayati E (2017). Family support and exclusive breastfeeding among Yogyakarta mothers in employment. Asia Pac J Clin Nutr.

[42] Angelo BHB, Pontes CM, Sette GCS, Leal LP (2020). Knowledge, attitudes and practices of grandmothers related to breastfeeding: a meta-synthesis. Rev Lat Am Enfermagem.

[43] Sihota H, Oliffe J, Kelly MT, McCuaig F. Fathers' experiences and perspectives of breastfeeding: a scoping review. Am J Mens Health. 2019;13(3). 10.1177/1557988319851616. 10.1177/1557988319851616 PMC6537273 31092114

[44] Alianmoghaddam N, Phibbs S, Benn C (2017). New Zealand women talk about breastfeeding support from male family members. Breastfeed Rev.

[45] Pacheco F, Sobral M, Guiomar R, de la Torre-Luque A, Caparros-Gonzalez RA, Ganho-Ávila A (2021). Breastfeeding during COVID-19: a narrative review of the psychological impact on mothers. Behav Sci (Basel).

[46] Snyder K, Worlton G (2021). Social support during COVID-19: perspectives of breastfeeding mothers. Breastfeed Med.

[47] Zanardo V, Tortora D, Guerrini P, Garani G, Severino L, Soldera G, Straface G (2021). Infant feeding initiation practices in the context of COVID-19 lockdown. Early Hum Dev.

